# Lockdown impact on age-specific contact patterns and behaviours, France, April 2020

**DOI:** 10.2807/1560-7917.ES.2021.26.48.2001636

**Published:** 2021-12-02

**Authors:** Paolo Bosetti, Bich-Tram Huynh, Armiya Youssouf Abdou, Marie Sanchez, Catherine Eisenhauer, Noémie Courtejoie, Jérôme Accardo, Henrik Salje, Didier Guillemot, Mathieu Moslonka-Lefebvre, Pierre-Yves Boëlle, Guillaume Béraud, Simon Cauchemez, Lulla Opatowski

**Affiliations:** 1 Institut Pasteur, Université de Paris, Mathematical Modelling of Infectious Diseases Unit, CNRS UMR 2000, Paris, France; 2 Institut Pasteur, Epidemiology and Modeling of Antibiotic Evasion unit, France; 3 Université Paris-Saclay, UVSQ, Univ. Paris-Sud, Inserm, CESP, Anti-infective evasion and pharmacoepidemiology team, Montigny-le-Bretonneux, France; 4 Data Management Core Facility, Institut Pasteur, Paris, France; 5 DREES, French Ministry for Health, Paris, France; 6 Insee (The French National Institute of Statistics and Economic Studies), Montrouge, France; 7 Department of Genetics, University of Cambridge, Cambridge, United Kingdom; 8 Association DataCovid, Paris, France; 9 Sorbonne Université, INSERM, Institut Pierre Louis d’épidémiologie et de Santé Publique, Paris, France; 10 Infectious diseases department, University hospital of Poitiers, Poitiers, France

**Keywords:** Europe, France, public health policy, epidemiology, modelling

## Abstract

**Background:**

Many countries implemented national lockdowns to contain the rapid spread of SARS-CoV-2 and avoid overburdening healthcare capacity.

**Aim:**

We aimed to quantify how the French lockdown impacted population mixing, contact patterns and behaviours.

**Methods:**

We conducted an online survey using convenience sampling and collected information from participants aged 18 years and older between 10 April and 28 April 2020.

**Result:**

Among the 42,036 survey participants, 72% normally worked outside their home, and of these, 68% changed to telework during lockdown and 17% reported being unemployed during lockdown. A decrease in public transport use was reported from 37% to 2%. Participants reported increased frequency of hand washing and changes in greeting behaviour. Wearing masks in public was generally limited. A total of 138,934 contacts were reported, with an average of 3.3 contacts per individual per day; 1.7 in the participants aged 65 years and older compared with 3.6 for younger age groups. This represented a 70% reduction compared with previous surveys, consistent with SARS-CoV2 transmission reduction measured during the lockdown. For those who maintained a professional activity outside home, the frequency of contacts at work dropped by 79%.

**Conclusion:**

The lockdown affected the population's behaviour, work, risk perception and contact patterns. The frequency and heterogeneity of contacts, both of which are critical factors in determining how viruses spread, were affected. Such surveys are essential to evaluate the impact of lockdowns more accurately and anticipate epidemic dynamics in these conditions.

## Introduction

Following its discovery in Wuhan province, China in December 2019, the severe acute respiratory syndrome coronavirus 2 (SARS-CoV-2) has quickly spread around the world. On 17 March 2020, the French government implemented a national lockdown to attempt to contain the epidemic and avoid overburdening healthcare capacity. This lockdown included multiple measures such as school closures (except for children of essential workers), closures of universities, restaurants, non-essential shops and most workplaces. Working from home was promoted where possible. Outdoor physical activity was restricted to 1 h per day and no further than 1 km from home. To help ensure compliance, individuals had to fill in a form each time they left home, stating the reason from a limited list of possible options. Understanding how these unprecedented measures impacted population behaviour and contact patterns is important to better characterise SARS-COV-2 transmission dynamics during the lockdown period. The social, cultural and behavioural responses to disease, communication with the public through official or media channels and strict measures heavily shaped the evolution of the outbreak [1-3]. Mathematical models often rely on empirical data describing the rate at which individuals mix with each other according to their age. Although such data are available from surveys performed in multiple countries in a non-epidemic situation [4-6], limited information is available during exceptional events such as a national lockdown. Therefore, we developed a survey to quantify how the set of measures enforced by the government impacted the contact patterns and behaviours of the French population. Using these data, we constructed contact matrices, which are useful tools to evaluate past or future lockdown impact and anticipate the dynamics of the virus in the population.

## Methods

### Survey

We set up SocialCov [7], an online survey to record contact patterns and behaviours within the French population. The survey included two parts. The first part referred to the period between 10 April and 28 April 2020. Using convenience sampling, participants were asked about their contacts and behaviour for the preceding day. After completion of the first part, the participants were invited to complete a second part referring to their contacts on a day before the national lockdown. The survey was advertised on social networks including posts on Facebook and Twitter, on the Institut Pasteur website and circulated via email. All individuals aged 18 years and older were invited to fill in the questionnaire. Information collected included socio-demographic information: age, sex, place of residence, number and age of household members, employment characteristics and whether changes occurred in the place of residence, household composition and employment status during the lockdown.

Participants also reported information about the individuals they have been in contact with and their characteristics. These included age and the setting/type of contact, for example if the contact happened at the workplace, in public transport, during a medical appointment, during physical activity practices, during shopping, or during the assistance to a person in need on the previous day. A contact was defined as either a physical contact such as a kiss or a handshake, or a close contact such as face-to-face conversation at less than 1 m distance. Data regarding childcare arrangements, lifestyle habits (such as shopping frequency, mode of transport, etc.) and individual protective behaviours including hand washing and mask wearing were also collected. Participants were asked to provide this information for the previous day, which was during lockdown and on a typical day before the lockdown.

### Statistical methods

We grouped study participants and contacts into the following age groups: 18–20, 21–25, 26–30, 31–35, 36–40, 41–45, 46–50, 51–55, 56–60, 61–65, 66–70, 71–75, 76–80, 81–85, 86–90, > 90 years and computed descriptive statistics for the responses related to age group, household composition, location, work activity and individual preventive measures. We present either the distributions of numbers and percentages or means and standard deviations as appropriate. Because most fields of the questionnaire were compulsory, no imputation of missing data was necessary. For the fields that were not compulsory, we report the number of answers in the Supplementary Table S3.

#### Contact matrices

We computed the average number of contacts per person per day stratified by age group, sex, work activity and place of contact and built contact matrices. For home matrices, all individuals of a household were considered as contacts. For matrices for other settings such as for contacts at the workplace, during shopping and in public transport, we computed contacts and age of contacts by summing those reported by the participant in the corresponding setting. We specifically asked the participants to detail their contacts with people in other locations who were not members of their household.

Outliers (n = 94) were defined as participants reporting more than 100 contacts per day or more than 30 contacts per day outside of their work and household location. These outliers were removed from the analysis. A global matrix that reported the average total number of contacts per day across ages was also computed. In this matrix, for each age group, the average number of contacts was reweighted to account for sex and professional activity distributions in the French population during the national lockdown using the COVID-19 Barometer survey as outlined in the next section.

### Sources of data used for population correction

In order to limit bias attributable to the study design and potential lack of representativeness of the study population, estimates were corrected using metrics from two data sources.

We used demographic data of the French population reported by the French National Institute for Statistics and Economic Studies (Insee) to compute weighted estimates and reproduce the age and sex distribution in the French population of those aged 18 years and older [8]. Weighting was achieved for all estimates related to professional situation and lockdown-associated behaviours. No weighting was done to compute descriptive statistics related to the survey population.

We also used data from the COVID-19 Barometer [9], which is a separate survey carried out during the same study period (i.e. April 2020) by the non-profit organisation DataCovid. In this survey, weekly online polls were administered by the company Ipsos with samples of 5,000 people representative of the French metropolitan population aged 18 years and older established by the quota method (Supplement, Section B). Estimated professional activity distributions by sex were used as a reference measure to compute corrected estimates of the global matrix.

When presenting unweighted data, we use the term participant. When presenting weighted data, we use the term French population.

### Comparison of contact frequency during lockdown with pre-lockdown

We compared the frequency of contacts during the lockdown with the frequency during pre-lockdown period (a non-epidemic period). To do so, we used reports related to participants’ contacts on a day preceding the national lockdown in the SocialCov survey and data from the COMES-F survey [10].

Participants of the SocialCov survey were also asked to fill an additional pre-lockdown survey and to depict their usual number of contacts at the workplace for a day preceding the national lockdown. A total of 35,982 participants completed this second questionnaire. We used the full dataset to compute the distribution of the number of contacts. We considered only the participants who declared that they work outside home both in the pre-lockdown period and during lockdown (3,186 participants) to assess the individual change between the number of contacts before and during the lockdown.

The COMES-F study was the first French large-scale population survey performed by Ipsos in 2012 [10]. The aim of the study was to describe the mixing pattern by age in the French population. In total 2,033 participants reported their contacts over 2 days: on 1 weekday and on 1 weekend day. To match and compare the two datasets, individual contacts in SocialCov were censored at 40 daily contacts to comply with COMES-F constraints and we removed them from this specific analysis. In addition, only contacts during weekdays were considered for the COMES-F survey. Censoring all contacts ≥ 40 generated a reduction of 0.2 in the average number of contacts in our survey, from 3.3 without censoring to 3.1 with censoring.

Data sources used in the study are summarised in Table 1.

**Table 1:** Data sources used to analyse the lockdown impact on age-specific contact patterns and behaviours, France, April 2020

Data source	Reference	Type of data	Use
SocialCov pre-lockdown survey	[7]	Pre-lockdown survey of contacts for the same study population	Comparison to pre-lockdown contacts at work for the same population
COMES-F survey	[10]	Survey on contact patterns by age in the French population in 2012	Comparison to pre-lockdown contacts in the French population
Insee	[24]	Demographic data of the French population (age and sex)	Population correction for the global contact matrix and the lockdown associated behaviours
COVID-19 Barometer	[9]	Survey on preventive behaviours, compliance with French policies and recommendations among the population^a^	Population correction for the global contact matrix

COVID-19: coronavirus disease; Insee: The French National Institute of Statistics and Economic Studies.

^a^ Details provided in the Supplement, Section B.

### Ethical statement

Data were collected in accordance with the regulations in force in France and in the European Union for the protection and security of personal data. In this study, no directly identifying data was collected and it is not possible to re-identify individuals through cross-checking. Aggregated data are accessible online (https://zenodo.org/record/5704755#.YaEkLdDMKUl). The complete dataset can be provided on demand, subject to prior determination of the terms and conditions of the request and in respect of the compliance with the applicable regulation.

## Results

### Study population

A total of 42,036 participants completed the first part of the questionnaire and 35,982 participants completed both parts between 10 April and 28 April 2020, including 28,796 females and 13,240 males from across France (Supplementary Figure S1A). A total of 94 responses were outliers and discarded from further analyses. There was an average of 4.4 participants per 10,000 inhabitants ranging from 0.89 per 10,000 in Haute Corse to 29.5 per 10,000 inhabitants in Paris (Supplementary Figure S1B and Table S2).

Half of the participants were under the age of 45 years and 13% were over the age of 65 years (Supplementary Table S1). The average number of household contacts was the highest for participants in the age group of 18–20-year-olds with 2.9 contacts per day, subsequently dropping for those in their 20s to 2.0 and 1.4 contacts per day and increasing again to a second peak for individuals aged 41–45 years (2.5 contacts per day) (Supplementary Figure S1C). Participants over the age of 60 years had ca one household contact on average. While 37% of participants declared being locked down with children (under the age of 18 years) in the household, 2.5% of participants over the age of 60 years declared a contact under the age of 18 years at home, compared with an average of 77% for those in the age group of 41–45-year-olds (Supplementary Figure S1E). Most participants declared working from home during lockdown (n = 22,327) or being unemployed or retired (n = 15,142).

### Contacts and age stratified contact matrices

**Figure 1:**
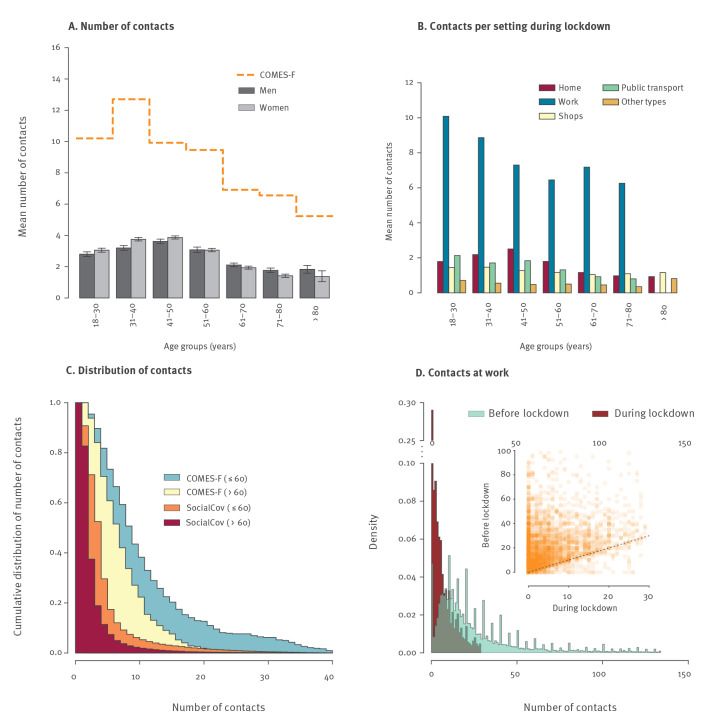
Number of contacts by age group and setting, during and before lockdown, France, April 2020

In total, 138,934 contacts were reported, representing an average of 3.30 contacts per day (median = 2; 2.5th to 97.5th percentile: 0–16, daily contacts) per participant after removing outliers. Individuals aged > 65 years reported on average 1.67 contacts per day compared with 3.55 contacts per day for those aged < 65 years (Figure 1A). These estimates are 70% (range: 61–76) lower than those measured in the COMES-F survey performed in France in 2012 (Figure 1A) [10].

A. Mean number of contacts depending on the age of participant. For each age group (x-axis), the bars represent the mean and 95% confidence interval (CI) of the number of contacts reported by participants, whatever the setting. The orange line represents the mean number of contacts for the COMES-F study [10]. To compare the two datasets, individuals with more than 40 contacts per day were not considered.

B. Mean number of contacts depending on the setting. For each age group (x-axis), bars represent the mean number of contacts for each setting reported by participants.

C. Light blue and yellow curves show the cumulative distributions of the number of contacts for participants in the COMES-F dataset aged 18–60 years and older than 60 years, respectively. Orange and red curves represent the cumulative distributions of the number of contacts for participants in the SocialCov study for participants aged 18–60 years and older than 60 years, respectively. To compare the two datasets, individuals with more than 40 contacts per day were not considered.

D. Distribution of the number of contacts at work before lockdown (grey), and during lockdown (red) only using observations up to the 95 percentile. The inset shows the change in the number of contacts before and during lockdown at the workplace for participants working outside their home during the lockdown (orange).

Furthermore, 4,567 (11%) participants reported contacts at work, 12,967 (31%) contacts in shops and 203 (0.5%) contacts in public transport. Finally, 12,325 (29%) participants reported contacts in other settings, which included medical appointments, physical activity practices and visits/assistance to relatives in need but with a much lower intensity than in other settings. The average number of daily contacts in each setting is shown in Figure 1B. The intensity of contacts was the highest at work, with the average number of contacts ranging from 6.3 for participants aged 71–80 years (the proportion of individuals in this age group declaring to work was limited, Supplementary Figure S2B) to 10.1 for participants aged 18–30 years. It was much lower in other settings, with between 0 and 2.5 contacts per day on average.

Critically, the proportion of individuals with a higher number of contacts markedly decreased during the lockdown (Figure 1C). Specifically, 4.9% of the participants aged 18–60 years reported more than 10 contacts per day compared with > 35% in the COMES-F study [10]. This percentage decreased to 1.7% among the people over the age of 60 years in our sample while it was 18.8% in the COMES-F study. Similarly, among those who maintained their professional activity outside home, after removing the top 5% to limit the impact of the tail of the distribution, the intensity of contacts at the workplace was reduced by 79% between the week preceding the lockdown and the lockdown period, from 25.4 to 5.4 contacts per day, with a distribution shifting towards lower values and approaching zero in the majority of cases during the lockdown (Figure 1D).

**Figure 2:**
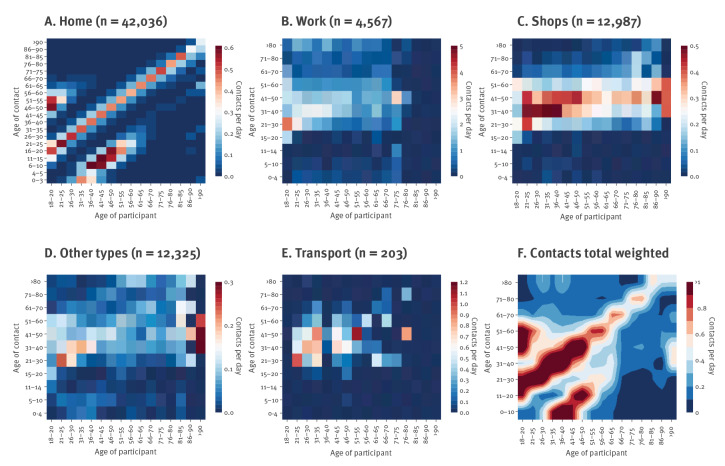
Contact matrices during national lockdown, France, April 2020

Using participants’ reports of their contacts across age groups, we built age-stratified contact matrices for each setting. The resulting home matrix is highly assortative by age group with a strong interaction between parents and children (Figure 2A).

Frequency of individual close contacts at less than 1 m per day according to age of participant (x-axis) and age of the contact (y-axis).

All contacts weighted using the French population distribution [8] and professional activity distributions during lockdown (COVID-19 Barometer).

In contrast, the contacts at work or in shops were less assortative (Figure 2 B-C), with an average number of daily contacts of 7.9 at work and 1.3 in shops for those who reported such contacts the previous day. The average number of contacts in public transport was low (computed based on 203 participants only) with an average of 1.7 contacts per day and representing a small fraction of the total number of contacts within the population (Figure 2E). Finally, the weighted global matrix depicts a higher intensity of within age group contacts and between-generation family-like contacts, consistent with previous findings [11,12]. Individuals aged > 65 years had limited contacts with the youngest age group (under the age of 20 years) and had on average 50% fewer daily contacts than individuals aged 30–55 years (Figure 1 and 2F).

In addition to contacts of study participants, some information on children’s contacts within and outside the household were available through participants’ reports. During lockdown, the majority of children's contacts occurred in the household. A total of 5% (n = 795) of participants with children had their children cared for outside home, with school or nursery attendance reported by 2% of participants. Only 3.4% (n = 508) of the children for whom contact information was reported had contacts with children that were not household members (Supplementary Figure S6), with an average of 2.5 contacts per day.

The total number of daily reported contacts also varied between geographic areas. In departments with > 500 participants, the average number of daily contacts varied between 2.7 (95% confidence interval (CI): 2.4–3.0) in Alpes-Maritimes and 4.4 (95% CI: 3.8–5.0) in Seine-Maritime. Participants from geographic areas with higher densities generally reported higher frequencies of contacts outside home and work (Supplementary Figure S7).

### Lockdown associated behaviour

**Figure 3:**
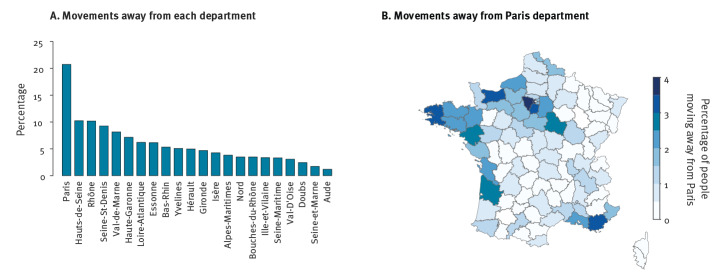
Spatial dispersion during national lockdown, France, April 2020

In our survey, Paris was by far the area that exhibited the highest level of migration, with ca 20% of participants from Paris declaring being located in another department during the lockdown (Figure 3A), predominantly in the West and South East of France (Figure 3B). Participants from Paris who declared being in a different geographic area reported a lower number of contacts outside home than those who stayed in Paris; 1.4 and 0.6 contacts per day on average, respectively (Supplementary Figure S8).

A. Percentage of the respondents for whom department of stay during lockdown differs from their home department. Only departments with more than 500 responses are represented in the figure.

B. Maps of department residencies during lockdown for individuals declaring department 75 (Paris) as their home department.

**Figure 4:**
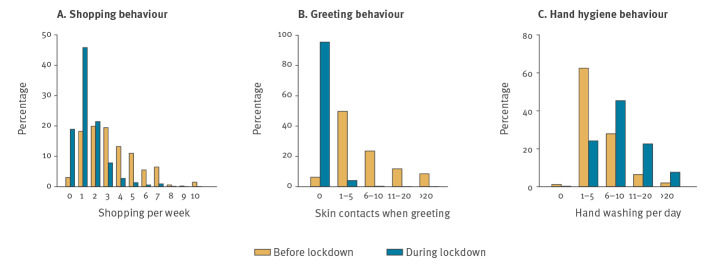
Lockdown-associated behaviours, France, April 2020

We estimated that < 2% of the French population used public transport during the lockdown, compared with 39% before the lockdown (Table 2). Shopping frequency also decreased. Approximately 60% of the population used to go shopping more than two times per week before the lockdown, whereas only 14% of the population went shopping during the lockdown. Interestingly, 19% did not visit any shops in the preceding week (Figure 4A).

**Table 2:** Changes in behaviour and risk perception during national lockdown, France, April 2020^a^

Survey questions	Response options	%
**Did you use public transport before and during lockdown?^b^**	Before lockdown	38.6%
	During lockdown	1.4%
**What would be your decision regarding a planned non-urgent medical appointment?**	Maintain appointment	19.2%
	Cancel or postpone appointment	48.4%
	Use telemedicine	32.4%
**Do you usually wear a mask outside of home?**	No	47.7%
	Sometimes	19.1%
	Yes	26.6%
	Not applicable	6.6%
**Would you wear a mask at home in case of symptoms?**	No	35.0%
	Sometimes	7.7%
	Yes	57.3%
**Risk perception. The outbreak represents...**	No risk	1.5%
	A risk for myself and my relatives	53.0%
	A risk for the elderly people	17.5%
	A risk for both	28.0%
**Has your risk perception changed since lockdown was implemented?**	No change	32.2%
	Increased	63.4%
	Decreased	4.4%

^a^ For each question, the percentage was computed based on a minimum of 40,907 and a maximum of 42,036 reports and weighted to account for the French population distribution (age and sex) reported by the French National Institute of Statistics and Economic Studies in 2019.

^b^ Participants were asked about the frequency of use of public transportation before and after the lockdown in two separate questions.

A. Change in shopping frequency. Distributions of shopping frequency (number of times individuals shop in a week) before (yellow) and during (blue) lockdown.

B. Frequency of hand washing before and during lockdown. Distributions of number of reported contact-associated to greeting behaviour in a typical day before (yellow) and during (blue) lockdown.

C. Change in greeting behaviour. Distributions of reported frequencies of hand washing in a typical day before (yellow) and during (blue) lockdown.

The percentages are weighted to account for the French population distribution (age and sex) reported by the French National Institute of Statistics and Economic Studies in 2019 [9].

The outbreak and lockdown affected risk perception and health-associated behaviours in the population. More than 60% of the French population declared that their perception of the risk associated with the outbreak had changed following the implementation of the lockdown measures (Table 2). Only 1.5% declared perceiving no risk associated with the outbreak and more than 80% of the population reported a perceived risk for themselves and their relatives (Table 2). In terms of barrier measures, the percentage of the French population washing their hands more than six times a day increased from 36% before the lockdown to 76% during the lockdown (Figure 4C). Greeting behaviour was also modified, with an estimated 95% of the population not physically greeting anyone outside the household by kissing or shaking hands, compared with ca 6% before the lockdown (Figure 4B). While 47% of the population declared that they did not consistently use masks outside home, 57% would wear one at home if symptomatic (Table 2). Finally, 81% of the population declared that they would not physically attend a non-urgent medical appointment: 48% would cancel the appointment and 32% would use telemedicine (Table 2).

After correction for population demographics, we estimated that during the lockdown, 44.2% of the French population aged 18 years and older were unemployed or retired (33.9% before the lockdown), 46.7% worked from home (3.7% before the lockdown) and 9.1% worked outside home (62.3% before the lockdown) (Supplementary Figures S2A and S2B). We estimated that among individuals who normally work outside home, 68.4% switched to telework and 16.8% became unemployed or retired (Supplementary Figure S2C).

## Discussion

The SocialCov study reveals a strong impact of the lockdown on social contact patterns and behaviours in France, with over 40,000 responses from people of diverse ages and across all of France. We demonstrate a major drop in contact frequency even for the more connected individuals and the substantial lockdown impact on population’s working conditions (working mode and employment status) and contact behaviours. The age-stratified matrices by setting also highlighted high assortativity by age group, with a strong interaction between parents and children and limited cross-generation contact with elderly people.

A substantial drop in contacts in all age groups was observed, including among individuals who maintained a professional activity outside home. Notably, both the mean and the dispersion of contacts were affected. The tail of the distribution of the daily number of contacts was strongly diminished, with less than 5% of the participants reporting more than 10 contacts per day. During the early pandemic phase, studies suggested a key role of superspreading events in the SARS-Cov2 outbreak [13,14]. In general, decreasing the number of contacts in the most socially active part of the population might have led to fewer superspreading events and less virus diffusion.

The reduction in contact frequency estimated in our study is consistent with estimates from other studies in Europe [15]. Jarvis et al. reported a 74% reduction in the average daily number of contacts, with an average of 2.8 contacts per day from 1,356 participants in the United Kingdom [11]. Similar reductions were observed in China, where on average two daily contacts were reported by 636 study participants in Wuhan and 557 participants in Shanghai, representing a reduction of 7–8-fold during the COVID-19 physical distancing period and with most interactions restricted to the household [12].

Participants reported an increased perception of risk about the pandemic after the introduction of the measures enforced by the government. The increased risk perception could also be seen as the main driver of spontaneous behavioural changes in the population to mitigate the spread of the pathogen [1,3]. During the lockdown, we registered an increase in the frequency of hand hygiene and a change in greeting behaviour. In addition, a majority of our participants declared that they would either cancel a planned medical appointment or use telemedicine rather than attending in person. This reduction in seeking care contributed to a 51% reduction in consultations with specialised physicians [16], a 40% reduction in general practice consultations and 48% reduction in emergency department admissions [17]. This was also observed among individuals with chronic disease where 51% of the respondents declared they cancelled at least one medical appointment and 16% reported using telemedicine services [18]. Although lockdown measures have been demonstrated to be effective in reducing the spread of SARS-CoV-2 in France [19,20], this comes at a high cost in terms of financial, social and indirect health consequences [21,22]. Estimating the impact of those will be critical to precisely evaluate the benefits and costs of such a strategy.

A proportion of participants reported being locked down in a different department than their home department (Figure 3). This was relevant for participants living in big cities such as Paris (20%) or Lyon (10%). Our results are consistent with the analysis of mobile phone data indicating that 11% of the registered residents and 23% of the total number of people present in Paris the night before the lockdown moved to another location [23] and with another study that reports the same pattern of migration from big cities in France [24]. Political elections took place in France on the weekend preceding the implementation of the national lockdown. This could have led some individuals from Paris (e.g. residents who are not registered on the electoral list in Paris) to travel back to their region of origin to vote. This phenomenon might also be related to pre-lockdown travel that was also observed in other countries such as China (from Wuhan to other regions), Italy (from the north to the south) [25], Spain (from Madrid to other regions) [26] where people moved to their second residence or hometown to avoid being quarantined in the city before the implementation of lockdown.

A small proportion of participants reported using masks outside home. However, mask usage started to be mandatory on public transport only at the easing of lockdown (11 May 2020) and was not mandatory at the time of the survey. In addition, these relatively small numbers may have been impacted by the limited availability of masks to the general population during the study period, when mask sales and distribution were restricted to healthcare professionals.

The data and results presented here should be considered in the light of the following limitations. First, participants were recruited online. As a consequence, the study population may not be a representative sample of the French population. Second, as frequently observed in these types of studies, two-thirds of survey participants were women. However, the work situation (Supplementary Figure S9) and contact matrices (Supplementary Figure S10) did not differ substantially between males and females. After the reweighting step, the distribution of household sizes (Supplementary Figure S3) globally matched the one reported by Insee in 2019 [27].

The survey population might be over-represented by employees, executives and individuals who had the opportunity to work from home. It also excluded populations less connected to social networks, smartphones or Internet. For example, people older than 65 years old represented only 13% of our study population while they account for 20% of the French population. We estimate that during the lockdown 53% of French people worked from home, a substantially higher figure than estimates from other sources such as Direction de l’animation de la recherche des études et des statistiques, or the COVID-19 Barometer. These respectively estimated, that 25% and 15% of the population teleworked. In order to counteract this overrepresentation, the global contact matrix was computed by weighting according to sex and employment characteristics and status (i.e. telework, unemployed, retired etc) estimated from the COVID-19 Barometer.

This article provides novel data on contact patterns during a national lockdown. Following relaxation of lockdown measures and the reopening of school and workplaces, intermediate and progressive changes in behaviour and contact patterns are expected to have occurred. The monitoring of contact patterns, risk perception and behaviours following the ease of the restrictions will be key to assess the dynamics of contact patterns over time. Since contact-patterns are strongly culture- and country-specific, establishing and extending similar surveys across the entire pandemic period will be critical.

From this survey, we estimated that the lockdown in France led to major reductions of contact patterns across all age groups, compatible with the estimated decrease in the reproduction number of SARS-CoV-2 [19]. The resulting matrices and data can be directly plugged into mathematical models of human-to-human transmission of SARS-CoV-2. They may also be useful to model and evaluate the impact of lockdowns in future epidemic waves of SARS-CoV-2 or in future pandemics. Monitoring how contact patterns and at-risk contacts evolve over time will be key to characterise the dynamics of the virus.

## Supplementary Data

4072000 Supplement
