# Supplementary material for: Lockdown impact on age-specific contact patterns and behaviours, France, April 2020
Source: Euro Surveill. 2021 Dec 2;26(48):2001636. doi: 10.2807/1560-7917.ES.2021.26.48.2001636 (PMC8641071; doi:10.2807/1560-7917.ES.2021.26.48.2001636)
Supplement: Supplement [file 20-01636_BOSETTI_Supplement.pdf]

Supplementary materials to the article: Lockdown impact on age-specific contact patterns and behaviours, France, April 2020

This supplementary material is hosted by *Eurosurveillance* as supporting information alongside the article “Lockdown impact on age-specific contact patterns and behaviours, France, April 2020”, on behalf of the authors, who remain responsible for the accuracy and appropriateness of the content. The same standards for ethics, copyright, attributions and permissions as for the article apply. Supplements are not edited by *Eurosurveillance* and the journal is not responsible for the maintenance of any links or email addresses provided therein.

Table of content

A. Demographics and weighting
B. Covid 19 Barometer from DataCovid
C. Childcare during Lockdown in France
D. Change in the contact pattern at the workplace during Lockdown
E. Contact patterns depending on the department
F. Work situation and contact matrices stratified on gender

A. Demographics and weighting

Population description

42,036 participants completed the full questionnaire between April 10 and April 28 (42,130 in total and 94 were classified as outlier reports), including 28,796 females and 13,240 males from across France (Figure S1A, Table S1), with an average of 4.4 participants per 10,000 inhabitants (range from 0.89 per 10,000 in Haute Corse to 29.5 per 10,000 inhabitants in Paris). The region of Paris Île de France, and departments of Doubs, Aude, Haute Garonne, and Herault were over-represented, with >10 participants per 10,000 inhabitants (Figure S1B, Table S2). Half of the participants were <45 years old and 13% were >65 years (Figure S1 and S3).The number of household contacts was the highest for participants aged 18-20, subsequently dropping for those in their 20s and increasing again to a second peak for individuals in their late 40s (Figure S1C). Participants >60 years old only had about 1 household contact on average. 37% of participants declared being locked down with children (<18 years old) in the household. Only 2.5% of participants aged >60 years old declared a contact aged <18 years old at home, compared with an average of 77% for those aged 41-45 (Figure S1E). Among participants reporting children, only 5% had their children cared for outside home, with school or nursery attendance reported only by 2% of participants. For the remaining 95% of participants with children, children were taken care of at home by the participants (Figure S6).

Figure S1. Description of the participant population under lockdown. a) Age distribution of participants; b) Spatial distribution of completed surveys. The maps indicates, for each department of mainland France, the number of completed surveys for which participant declared usual address; c) Average number of other members in the household during the lockdown according to age of participant; d) Household size distribution in the SocialCov participant population during lockdown (black); e) Number of participants declaring having children (<18 years old) household members during lockdown, according to participant's age.

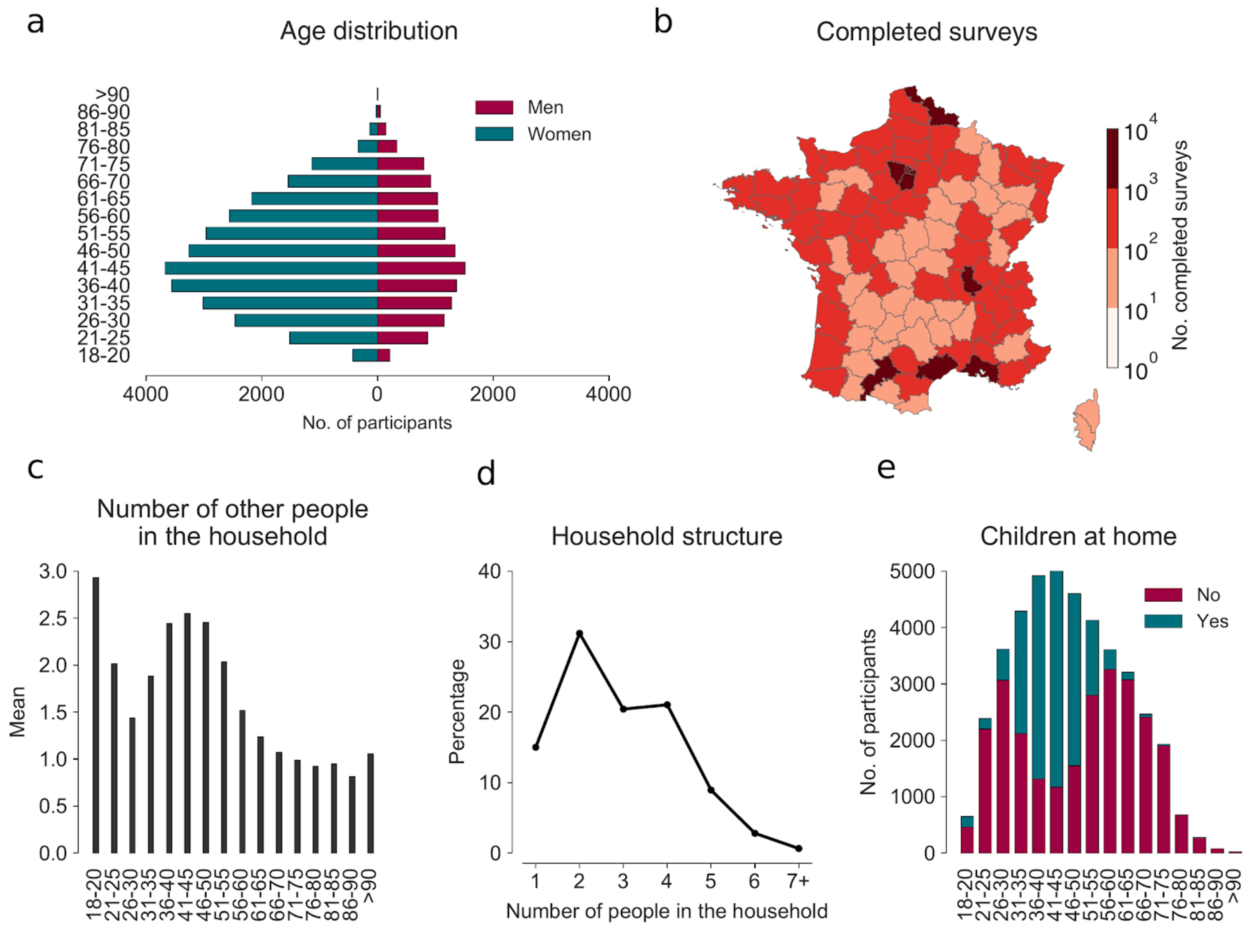

Table S1. Age of participants. Number of participants to the survey for each age class.

	Age class															
	**18 20**	**21 25**	**26 30**	**31 35**	**36 40**	**41 45**	**46 50**	**51 55**	**56 60**	**61 65**	**66 70**	**71 75**	**76 80**	**81 85**	**86 90**	**>90**
**Men**	216	867	1155	1277	1364	1510	1343	1164	1050	1035	923	798	335	145	49	9
**Women**	429	1518	2463	3018	3555	3672	3258	2964	2558	2176	1544	1133	337	135	27	9

Table S2.Number of completed surveys for overseas departments of France. For each overseas department of France, the number of completed surveys for which participants declared an usual address.

Overseas department	Number of participants

Guadeloupe	40
Martinique	26
French Guyane	14
La Réunion	145
Mayotte	4

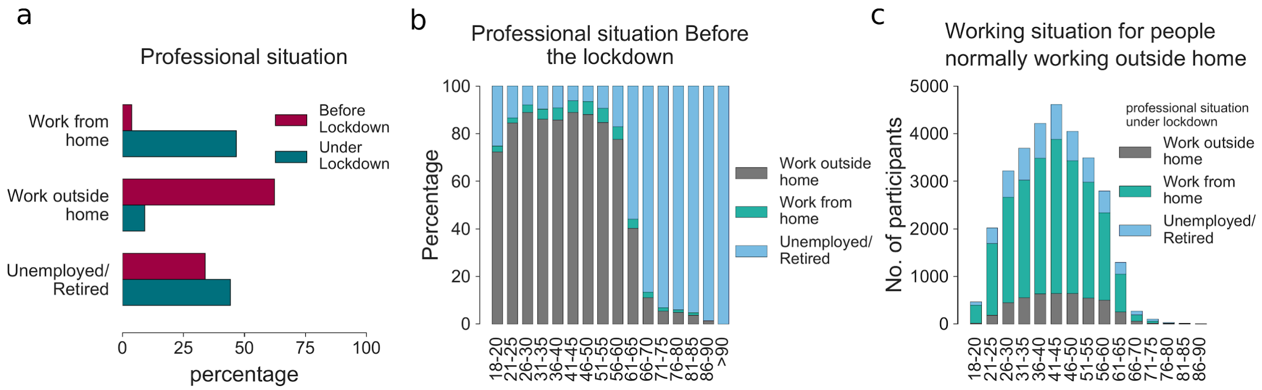

Figure S2. **Estimates of professional activity.** a) Distribution of professional situations before (purple) and during (blue) the lockdown weighted to account for the French population distribution reported in the INSEE in 2019[1]; b) Distribution of professional activities in the participant population (before lockdown); c) Professional situation under the lockdown for people working outside home before the lockdown.

Demographics

We compared the demographics of the SocialCov participating population with estimates from the French population by the National Institutes for statistics and Economic studies (INSEE). In SocialCov, 68% of participants were women with an overrepresentation of age-groups 41-45 which accounted for 12.33% and underrepresentation of the >65 (Figure S1). Age distribution did not change across France (Figure S4).

To correct for demography structure, the data was reweighted to account for the true French demography reported by INSEE in 2019 [1]. Both unweighted and weighted household structures from SocialCov had a similar trend than the one reported by INSEE in 2019-20 [2](Figure S1D and S3).

Figure S3. Household size distribution. Household size distribution in the SocialCov participant population: during lockdown (black full line) and weighted to account for the French population distribution reported by INSEE in 2019 [1](black dotted line). Household size distribution reported by INSEE From the second trimester of 2019 to the first trimester of 2020[2](red full line).

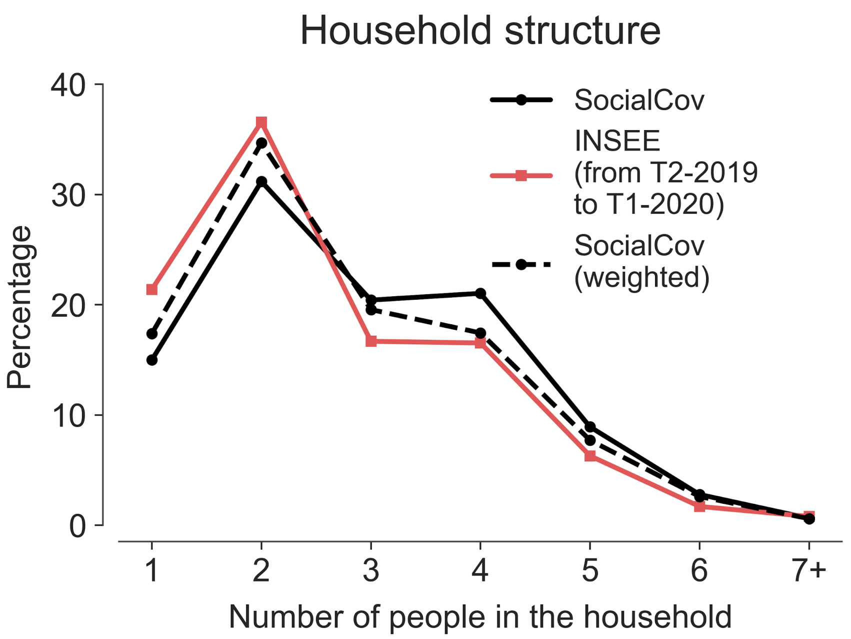

Figure S4. Age distributions of participants by department. Only departments with >500 participants are presented.

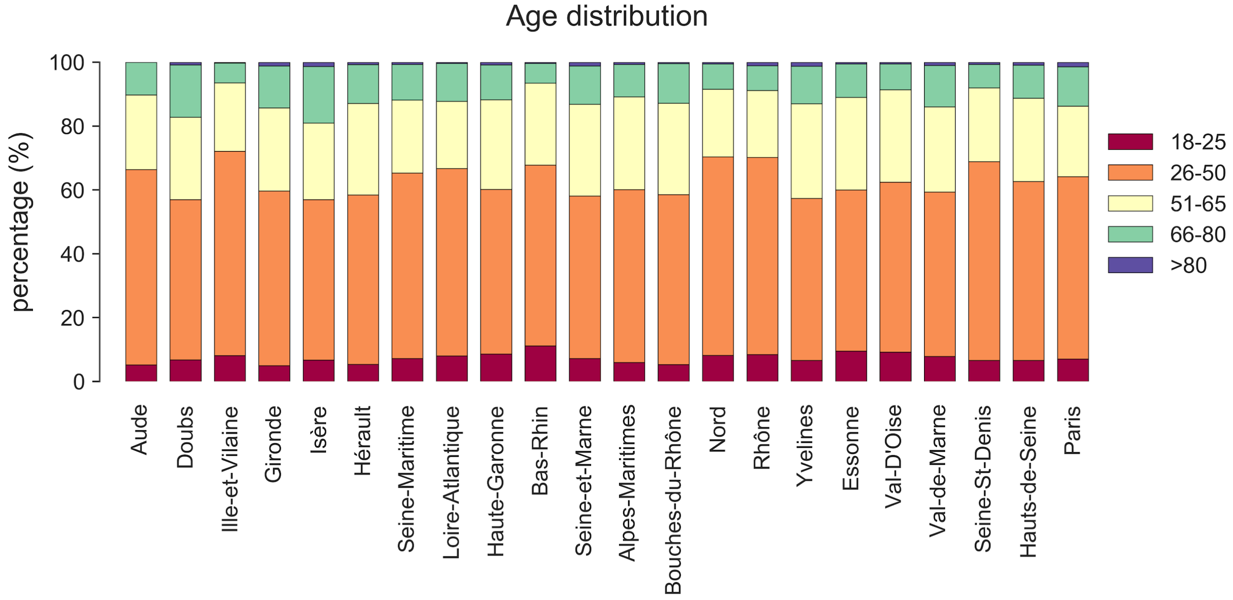

Figure S5. Impact of lockdown on professional activities of participants by departments. Distribution of professional activities during lockdown among participants who declared working outside home in normal times. Only departments with >500 participants are presented. Grey: work outside home; Green : work from home; Light blue : unemployed or retired.

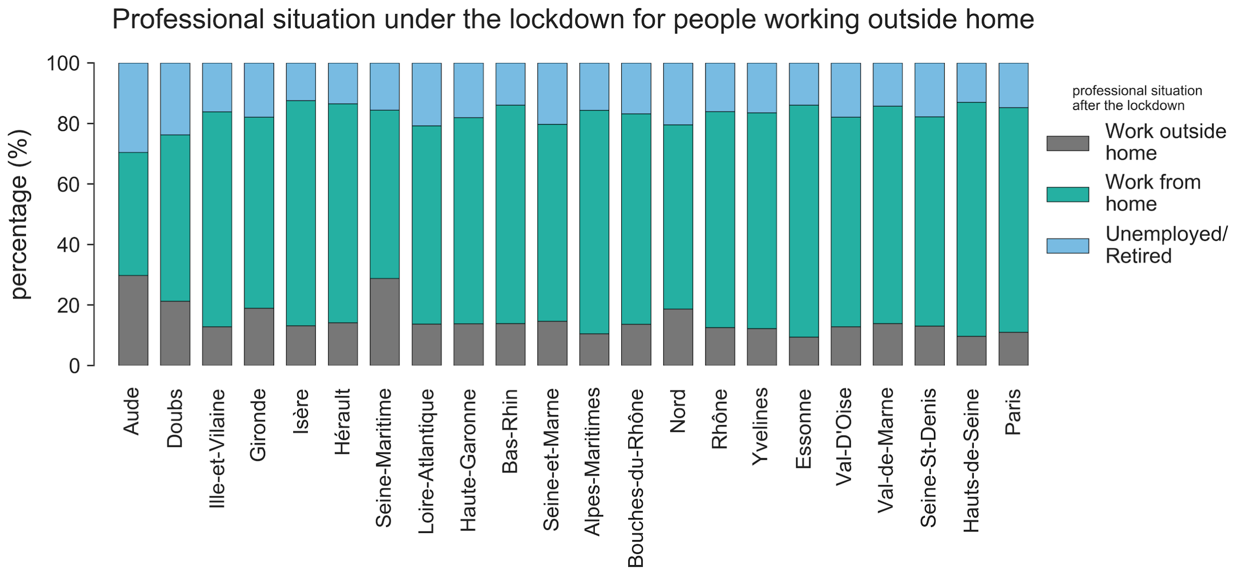

B. Covid 19 Barometer from DataCovid

The Covid19 Barometer, authored by the © COVID 19 Barometer contributors, is a citizen science initiative coordinated by the non-profit organisation DataCovid (https://datacovid.org/) that provides open access data from a weekly survey measuring SARS-Cov-2 dynamics, its determinants, and its impacts. In particular, the barometer provides longitudinal information relating to the symptoms of the infection, preventative behaviors, compliance with French policies and recommendations, and the socio-demographic, economic and psychological characteristics of the respondents. The weekly survey encompasses 5,000 respondents aged 18 or over and representative of the general French population. The barometer is operated online by Ipsos using its Access Panel and is funded by Gilead, Amgen, Johnson & Johnson, Roche, CNP Assurances, Vinci Autoroutes, RATP, and EDF. The quota method is employed based on the following variables: sex, age, urban area category, region, and profession of the respondent. The data are available under the "OPEN LICENSE 2.0" (https://datacovid.org/copyright/). For this study, data of waves 1, 2, and 3 were used.

Given its representativeness and comprehensive demographic data, the Covid 19 Barometre provides a valuable resource to estimate professional activity patterns in the French population. Here, we use these estimates for reweighting of our estimated contact matrices to adjust biases in the professional status of the survey population.

C. Childcare during lockdown

Participants were asked, when they had children <18 years old at home, to provide information about childcare at the time of the survey. Most of them (95%) declared that children were cared for at home, mostly by either the participant or another household member (Figure S6).

Figure S6. Who takes care of children during lockdown? Distribution of child caregiver reported by SocialCov participants. Children were either cared for at home by the survey "Participant", an "Other member of the household" or an "External person", or outside home, at "School/nursery". "Other" corresponds for instance to teenagers under 18 years old who didn't have any adult supervision.

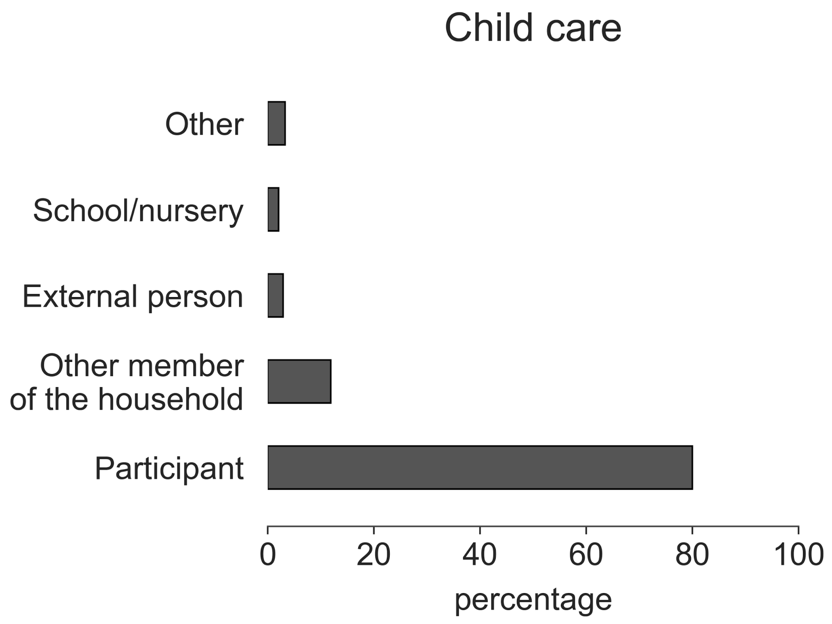

D. Contact patterns depending on the department

Figure S7. Contacts of participants by department, and correlation with department densities. A. Mean number of daily contacts (r^2= 0.05). B. Mean number of contacts excluding contacts with household members; all participants (red, r^2= 0.48); only participants who went out at least once in the past 24h (blue, r^2= 0.50). Only departments with >500 participants are presented. Average number of contacts and standard error of the mean are shown, data not corrected.

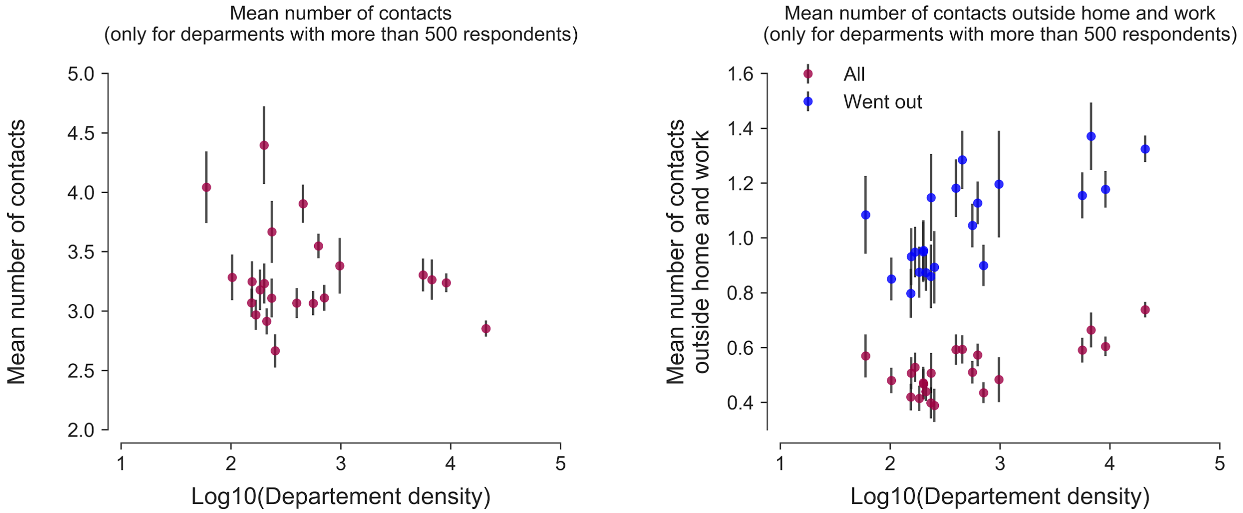

Figure S8. Average number of contacts of Paris inhabitants, comparison between those who stayed and those who moved to a different place during lock down. Both graphics indicate the mean number of contacts for Paris inhabitants declaring being locked down in Paris and those declaring being in a different department . Left: Mean number and 95%CI of daily contacts with others than household members. Right: Mean number and 95%CI of household members, data not corrected.

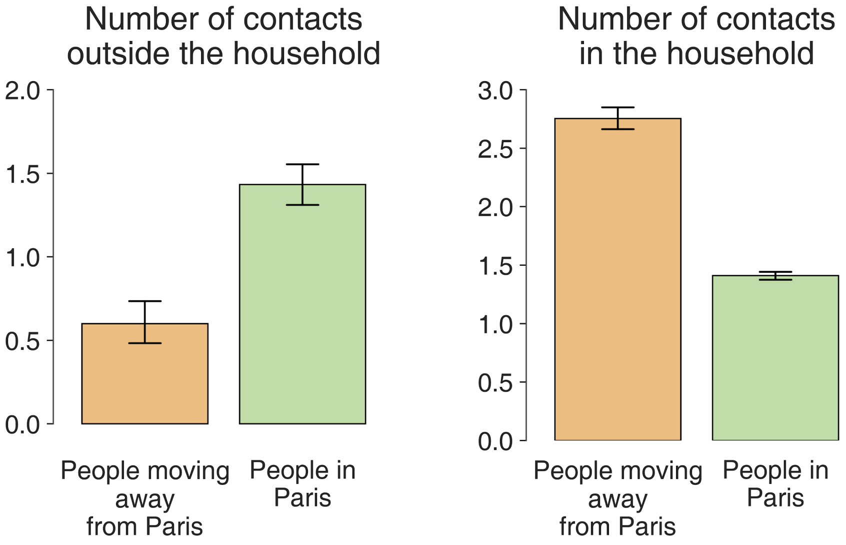

E. Work situation and contact matrices stratified on gender

Figure S9. Professional situation of participants by gender before and during lockdown . Top: men. Bottom: women. From left to right: A. Distribution of professional situations of participants before (purple) and during (blue) lockdown weighted to account for the French population distribution reported by INSEE in 2019. B. Distribution of professional activities and location of activity by age before lockdown (outside home or at home). C. Distribution of professional activities during lockdown by age among participants who declared working outside home in normal times. Grey: work outside home; Green : work from home; Light blue : unemployed or retired.

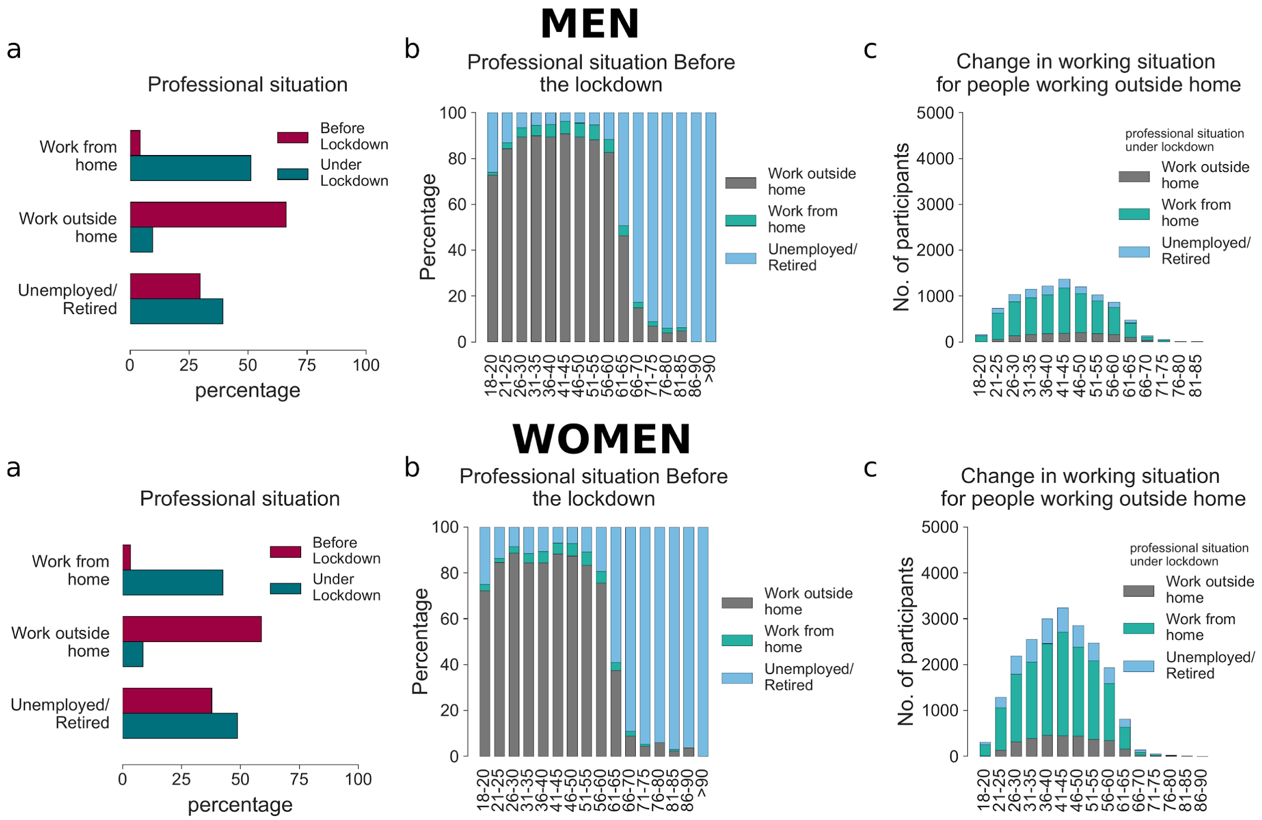

Figure S10. Contact matrices during lockdown in France, stratified by gender. Top: men. Bottom: women. Frequency of individual close contacts at less than 1 meter per day according to age of participant (x-axis) and age of the contact (y-axis). A. Contacts occurring at home. B. Contacts reported at the workplace. C. Contacts reported in shops. D. Contacts on other locations. E. Contacts in public transports. F. All contacts.

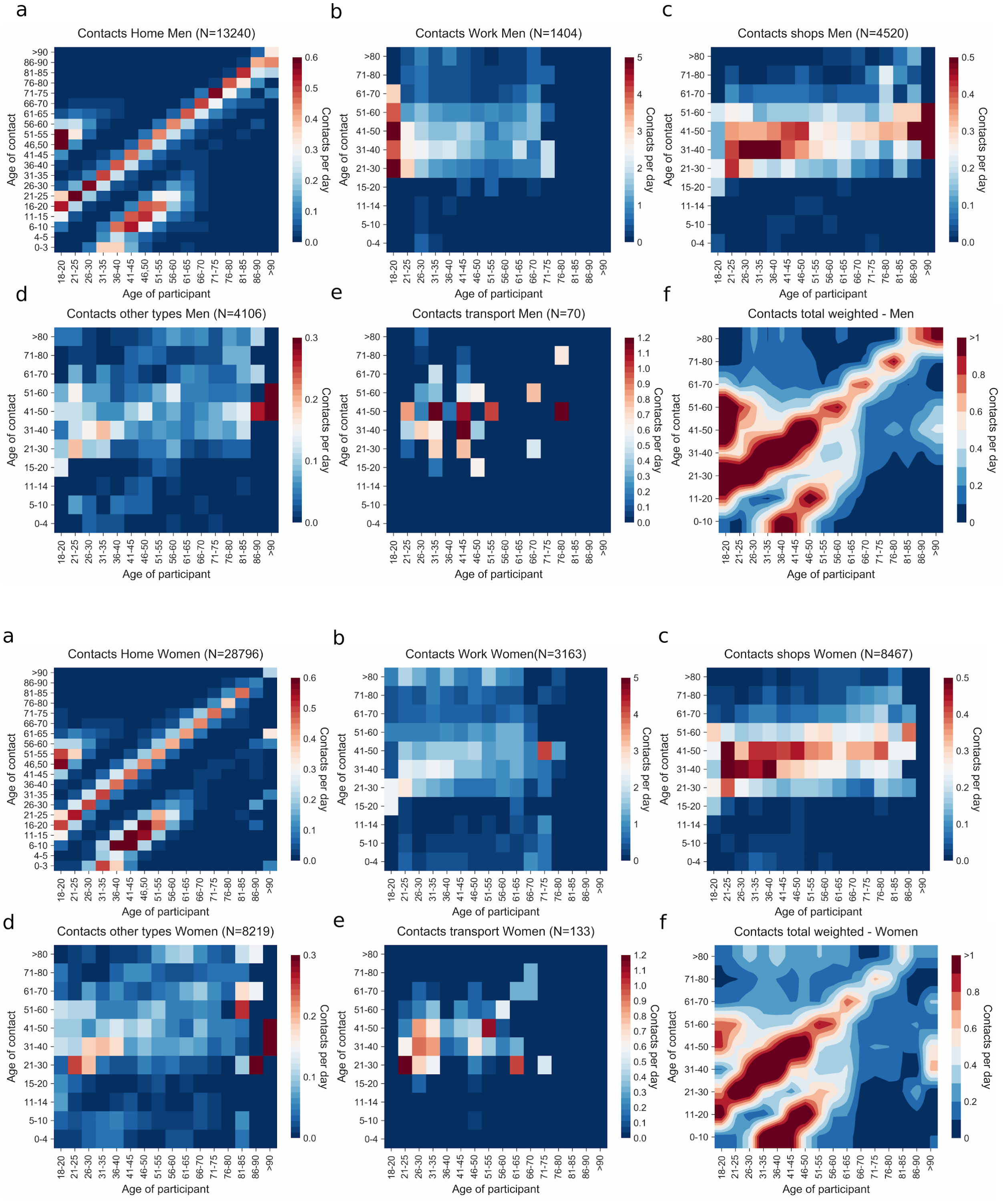

Table S3. Changes in behaviour following lockdown in France, uncorrected data. For each question, the % among the respondents of the question is provided. Number of respondents ranged 40,907-42,036 depending on the question

	Before the ban	During Lockdown
Frequency public transport (number of transports a day)	0: 61.6%<br>1: 4.4%<br>2: 23.7%<br>>2: 10.3%<br>n=41336	0: 98%<br>1: 0.4%<br>2: 0.6%<br>>2: 0.2%<br>n=40907
Frequency shopping (per week)	0: 1.8%<br>1: 17.9%<br>2: 19.8%<br>>2: 60.5%<br>n=41700	0: 16.7%<br>1: 49.3%<br>2: 21.5%<br>>2: 12.5%<br>n=41570
Frequency washing hands (per day)	0: 1%<br>1-5: 58.5%<br>6-10: 30.5%<br>11-20: 7.6%<br>>20: 2.4%<br>n=41,952	0: 0.2%<br>1-5: 21.6%<br>6-10: 45%<br>11-20: 24.4%<br>>20: 8.8%<br>n=41,499
Frequency kissing & shaking hands (per day, excluding household members)	0: 5.9%<br>1-5: 49.1%<br>6-10: 24.0%<br>11-20: 12.3%<br>>20: 8.7%<br>n=41,557	0: 95.9%<br>1-5: 3.6%<br>6-10: 0.3%<br>11-20: 0.1%<br>>20: 0.1%<br>n=41,786
Decision regarding planned non urgent medical appointment		Maintain appointment: 17.4%<br>Cancel or report app.: 48.8%<br>Use telemedicine: 33.8%<br>n=41,790
Wear a mask outside home		No: 48.4%<br>Sometimes: 20%<br>Yes: 26.7%<br>Not applicable: 4.9%<br>n= 41,939
Wear a mask at home in case of symptoms		No: 33.5%<br>Sometimes: 7.5%

		Yes: 59%<br>n= 41,880
Risk perception, the outbreak represents....		no risk: 1.3%<br>A risk for myself and my relatives”: 52.7%<br>A risk for the elderly”: 16.4%<br>A risk for both: 29.2%<br>n=42,036
Evolution of risk perception		No change:30.8%<br>Increase: 64.8%<br>Decrease: 4.4%<br>n=41,857

References

1. Age structure of the population − Demographic balance sheet 2019 | Insee. [cited 10 Jul 2020]. Available: https://www.insee.fr/en/statistiques/2382609?sommaire=2382613

2. Statistical presentation − Labour force survey 2019 | Insee. [cited 10 Jul 2020]. Available: https://www.insee.fr/en/metadonnees/source/operation/s1490/presentation
